# End-to-End Lip-Reading Open Cloud-Based Speech Architecture

**DOI:** 10.3390/s22082938

**Published:** 2022-04-12

**Authors:** Sanghun Jeon, Mun Sang Kim

**Affiliations:** Center for Healthcare Robotics, Gwangju Institute of Science and Technology (GIST), School of Integrated Technology, Gwangju 61005, Korea; jeon7887@gist.ac.kr

**Keywords:** audio-visual speech recognition, lip-reading, application programming interface, multi-modal interaction, deep neural networks

## Abstract

Deep learning technology has encouraged research on noise-robust automatic speech recognition (ASR). The combination of cloud computing technologies and artificial intelligence has significantly improved the performance of open cloud-based speech recognition application programming interfaces (OCSR APIs). Noise-robust ASRs for application in different environments are being developed. This study proposes noise-robust OCSR APIs based on an end-to-end lip-reading architecture for practical applications in various environments. Several OCSR APIs, including Google, Microsoft, Amazon, and Naver, were evaluated using the Google Voice Command Dataset v2 to obtain the optimum performance. Based on performance, the Microsoft API was integrated with Google’s trained word2vec model to enhance the keywords with more complete semantic information. The extracted word vector was integrated with the proposed lip-reading architecture for audio-visual speech recognition. Three forms of convolutional neural networks (3D CNN, 3D dense connection CNN, and multilayer 3D CNN) were used in the proposed lip-reading architecture. Vectors extracted from API and vision were classified after concatenation. The proposed architecture enhanced the OCSR API average accuracy rate by 14.42% using standard ASR evaluation measures along with the signal-to-noise ratio. The proposed model exhibits improved performance in various noise settings, increasing the dependability of OCSR APIs for practical applications.

## 1. Introduction

Automatic speech recognition (ASR) uses algorithms implemented in devices such as computers or computer clusters to convert voice signals into a sequence of words or other linguistic entities [1,2]. Previous ASR applications were based on interactive voice response, device control by voice, content-based voice audio search, and robotics. However, ASR technology has improved significantly in recent years owing to the exponential increase in data and processing power, which makes it possible to perform difficult applications. Voice search using mobile devices, voice control in home, and numerous speech-centric information processing applications that benefit from the downstream processing of ASR outputs are some of the examples of advancements in ASR technology [3]. Thus, noise robustness has become an essential core technology for large-scale, real-world applications given that OCSR APIs must exhibit improved functionality because of the significantly demanding acoustic scenarios.

**Figure 1:**
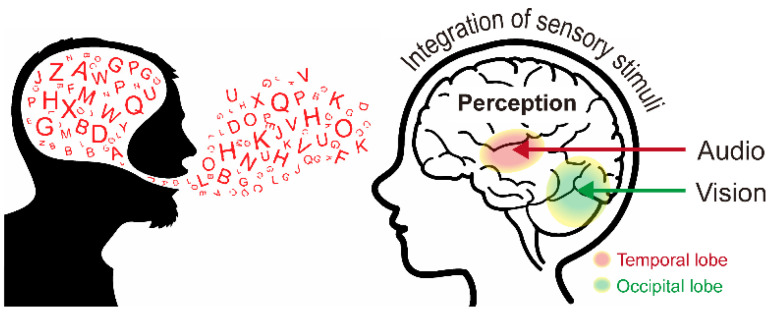
Multisensory integration comprising both auditory and visual information.

In this study, we propose a noise enhancement system that uses a multimodal interaction approach based on multisensory integration that refers to the interplay of information from several senses. Multisensory integration often influences the perception of human speech. The human nervous system comprises several specialized sensory organs, each of which conveys certain sensory information [4]. The organization of sensory organs in the human body is advantageous considering that each organ serves as a non-redundant source of information, which allows organisms to detect critical sensory events with higher certainty by separately examining the input received from each sense. However, when several sources of information are merged, information from different senses can be linked [5], and this can synergistically influence the capacity to notice, assess, and start reactions to sensory events (Figure 1) [6]. The brain is divided into four lobes; Figure 1 shows the audio (red, temporal lobe) and visual (green, occipital lobe) information in multisensory integration.

Several studies have shown that the contemporaneous observation of visual speech, such as the movement around the speaker’s lips, significantly affects speech perception. Visual speech information improves the ability to understand speech in scenarios when words are spoken with an accent, or when the surrounding environment is noisy [7,8,9]. For example, lip-reading can substantially improve the understanding of speech if the audio signal is unclear [10,11,12]. The McGurk effect illustrates how mismatched auditory and visual speech information affects speech perception [13]. For example, when we hear the sound “ba” while seeing a person’s face express “ga”, many people hear “da”, a third sound that is a combination of the two. This fusion approach contributes to the robustness of speech detection in a variety of real-world applications, such as human–machine interaction, by overcoming the problems of noise, auditory ambiguity, and visual ambiguity.

To the best of our knowledge, this is the first study that proposes a noise-robust OCSR API system based on an end-to-end lipreading architecture for practical applications in various environments. This system exhibits performance superior to those systems that comprise only audio or visual speech recognition technology. For auditory-based speech recognition, we evaluated the performance of four OCSR APIs (Google, Microsoft, Amazon, and Naver) using Google Speech Commands Dataset v2 and the collected dataset prepared by us to select the best API for our design. The word lists recognized in the highest-performance API were expressed as word vectors using the Google Word2Vec model, which was trained using the dataset of 1,791,232-word sentences. Similarly, we developed a new end-to-end lipreading architecture comprising two end-to-end neural subnetworks for visual-based speech recognition. The feature extraction method consists of the following components: a 3D convolutional neural network (CNN), 3D dense connection CNN for each time step to reduce the number of model parameters and avoid overfitting, and multilayer 3D CNN to capture multichannel information in the temporal dimension of the entire video to overcome insufficient visual information and obtain specific image features. A bi-directional gated recurrent unit (GRU) with two layers, followed by a linear layer, was used in the sequence processing module. Therefore, the integrated values of word vectors obtained from speech API and the integrated values of vectors obtained from the lip-reading model were concatenated to form vector matrix. After introducing a SoftMax layer at each time step with a concatenated vector matrix, the entire network was trained using the connectionist temporal classification (CTC) loss function to obtain probabilities. Furthermore, we compared the proposed system’s accuracy and efficiency with those of existing standalone techniques that extract the visual features and several OCSR APIs for the collected datasets. An extensive assessment revealed that the proposed system achieved an excellent performance and efficiency. Thus, we propose a noise-robust open cloud-based speech recognition API system based on an end-to-end lip-reading architecture for practical applications.

The remainder of this paper is organized as follows. Section 2 examines relevant research on OCSR APIs and visual speech recognition VSR systems. Section 3 introduces the architecture of the proposed system. Section 4 presents information on the benchmark datasets, custom collected datasets, audio-visual information processing, augmentation technique, experimental setup, and evaluation. Finally, Section 5 presents the discussion and conclusions.

## 2. Related Work

Google has published “Google Assistant” (an AI voice recognition assistant) and various speech recognition features for autos and consumer electronics as an open API. The technology, which supports more than 120 languages, includes various features, such as automatic punctuation, speaker distinction, automatic language identification, and enhanced voice adaptability. As part of Watson’s AI service package, which currently handles 11 languages, the company offers an ASR service. However, the custom acoustic and language model desired by the user must be initially trained using user data. In other words, to use a personalized acoustic model, the user’s own audio must be used, and a new corpus must be added to expand the language model. Further, Microsoft offers a cloud service package known as Azure, and speech-to-text is an API for speech recognition provided by Azure’s cognitive services. According to the official website, Azure employs “breakthrough voice technology” based on decades of study. Furthermore, their website alludes to a 2017 publication where Microsoft achieved the first-ever human-level accuracy on the switchboard test [14]. Alexa by Amazon is a voice-activated artificial intelligence (AI) smart personal assistant that includes features such as voice interaction and the ability to ask and answer questions. Further, Alexa can control smart gadgets featured in intelligent home technology. Alexa is easily available on Amazon Echo, Echo Dot, Echo Plus, and other smart speakers. In South Korea, the Naver collaboration aggressively developed speech recognition by launching Clova speech recognition (CSR) on 12 May 2017 [15]. The CSR now supports Korean, English, Japanese, and Chinese languages, although Korean has a better recognition rate.

Deep learning technology has recently demonstrated remarkable performance in a variety of applications, including VSR systems. Deep learning algorithms can achieve higher accuracy compared to older approaches with traditional predictions. For example, when a CNN is used in combination with conventional approaches, the CNN architecture can differentiate different visemes, and temporal information is added after obtaining CNN output using an HMM framework [16,17]. Furthermore, other studies [18,19] have integrated long short-term memory (LSTM) with histograms of oriented gradients (HOGs) and used the GRID dataset to input recognized short words. Similarly, word predictions were generated using an LSTM classifier with a discrete cosine transformation (DCT) trained with the OuluVS and AVLetters datasets [17]. The sequence-to-sequence model (seq2seq) is a deep speech recognition architecture that can read and predict the output of an entire input sequence. For longer sequences, it takes advantage of global information. These studies [20,21] demonstrated the recognition of audio-visual speech in a dataset based on real words using the first seq2seq model that incorporates both audio and visual information. The initial model is LipNet to use the end-to-end model and be trained using sentence-level datasets (GRID corpus) for performance evaluation [22]. The overlapped and unseen-speaker databases had word error rates of 4.8% and 11.4%, respectively, whereas human lip-readers had a success rate of 47.7% for the same database. In [23,24,25], digit sequence prediction using 18 phonemes and 11 terms, and other similar architectures such as the CTC cascade model were implemented to evaluate the convergence of audio-visual features. Therefore, deep learning techniques can extract more detailed information from experimental data, which demonstrates their high level of resiliency against big data and visual ambiguity.

Several previous studies [26,27,28] focused on evaluating the recognition performance of existing disclosed OCSR APIs and aimed to apply them to applications such as robots. However, this study focused on improving the system using visual information in the existing OCSR API system and showed a low error rate in a noisy environment; as a result, a high recognition performance was demonstrated. This study presents a model that achieves a low error rate even in noisy environments using deep learning-based audio and visual information compared to the existing OCSR APIs that rely solely on audio information.

## 3. Architecture of the Proposed Model

**Figure 2:**
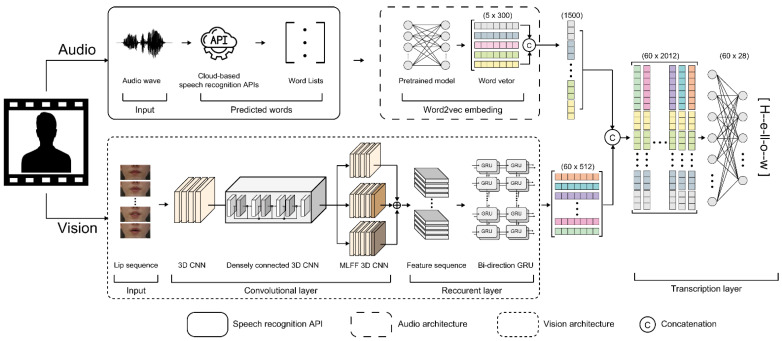
Block diagram of proposed audio-visual speech recognition system.

Our lipreading system is combined with existing cloud-based speech recognition systems, and the proposed audio-visual speech recognition system is shown in Figure 2. The audio architecture among Microsoft, Google, Amazon, and Naver was compared to select and combine the best performing OCSR API. The vision architecture of the proposed model was combined with the following feature extraction methods: LipNet (used as the baseline method), LeNet-5, Autoencoder, ResNet-50, DenseNet-121, and multi-layer CNN, all of which exhibit exceptional feature performance.

In all speech recognition engines, the user’s voice is transmitted to the recognition system using a microphone (Figure 2). To this end, we used two generic algorithms. The voice was processed on a local device, and the recorded voice was forwarded to a cloud server provided by Google or Microsoft for additional processing. Microsoft Cortana and Google are commercial engines that simultaneously separate speech recognition systems into closed and open-source code systems [29]. Speech recognition in OCSR APIs, which is a type of closed source, allows the rapid and easy construction of application speech recognition systems. Application speech recognition systems can be developed easily using OCSR APIs and are therefore gaining traction in a variety of sectors. Thus, developers of application speech recognition systems need to select suitable OCSR APIs based on the function and performance of the system. Furthermore, the performance of OCSR APIs varies depending on the date of the research and the type of learning data. Words output from the OCSR API are represented by word vectors using Google’s pre-trained Google’s Word2Vec model. Word2Vec is a set of shallow neural network models developed by Mikolov et al. to build “high-quality distributed vector representations that capture a large number of exact syntactic and semantic word associations” [30,31]. The dimensions of these word vector representations, also known as word embeddings, can be in the hundreds. To represent a document, word embeddings can be concatenated. Google has provided a Word2Vec model that has been pre-trained on the 100 billion words in the Google News corpus, resulting in 3 million 300-dimension word embeddings for academics. Therefore, five-word list were outputted and converted into 300-dimensional vectors and summed into one single vector (Figure 2).

**Figure 3:**
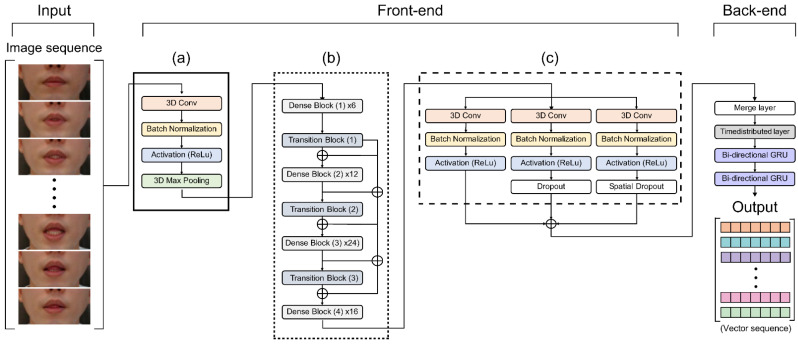
Proposed VSR system architecture. (**a**) 3D CNN; (**b**) 3D dense connection CNN; (**c**) multi-layer 3D CNN.

As mentioned above, we present a deep-learning-based VSR architecture and propose a new feature extraction method (Figure 2). Figure 3 and Supplementary Table S1 shows the detailed hyperparameters of the proposed architecture.

### 3.1. Convolutional Layer

**Figure 4:**
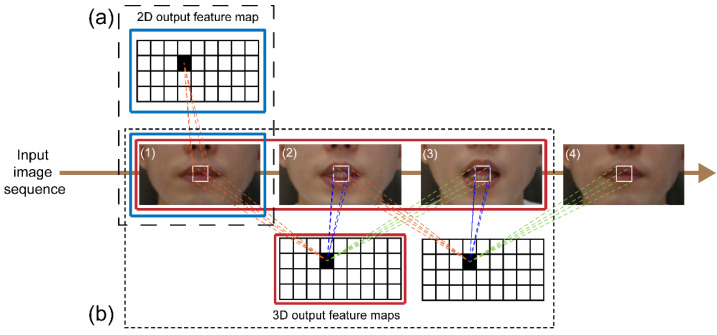
Comparison of convolutions in (**a**) 2D; (**b**) 3D.

CNNs use raw input data directly, which results in the automation of the feature development process. For image recognition, a 2D CNN is used to collect encoded information for a single picture dataset and to convert that information to 2D feature maps for computing features from spatial dimensions. However, the motion information contained in numerous contiguous frames fails when utilizing a 2D CNN for video recognition (Figure 4a). We used a spatial-temporal 3D CNN to calculate spatial and temporal features to capture distinct lip-reading actuations around the lips, such as tongue and teeth movements. When spatial and temporal information from following frames is considered, 3D CNNs have been found to be effective in extracting attributes from video frames in several experiments [16,22] (Figure 4b).

In this experiment, all consecutive frames input to encode the visual information of the lips were transmitted to the CNN layers in 64 3D kernels with a size of 3 × 7 × 7 to obtain feature information, as shown in Figure 3a. We reduced the internal covariate transformation using a batch normalization (BN) layer and ReLU to accelerate the training process. Additionally, a max-pooling 3D layer was added to reduce the spatial scale of the 3D feature maps (Supplementary Figure S1a).

A dense connection CNN generates relationships between multiple connected layers, allowing for full feature usage, vanishing gradient, and network depth. The input features are decreased by the bottleneck layer placed prior to the convolution layer. As a result, following the bottleneck layer operation, multichannel feature volumes are fused. Because the previous features are still present, the subsequent layer is only applied to a small number of feature volumes. Transition layers are also incorporated to increase model compactness due to the hyperparameter theta that controls the degree of compression. A decreased growth rate was achieved by using bottleneck and transition layers, resulting in a narrower network, reduced model parameters, efficiently controlled overfitting, and reduced processing resources.

**Figure 5:**
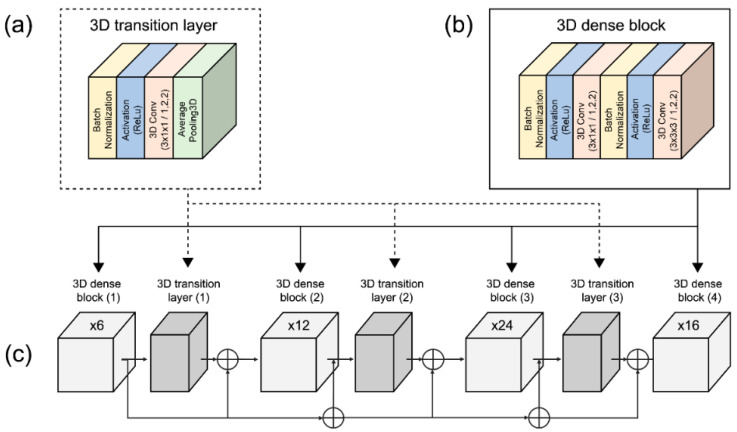
3D dense connection CNN architecture. (**a**) 3D transition layer structure; (**b**) 3D dense block structure; (**c**) detailed 3D dense connection CNN.

We implemented the 3D dense connection CNN architecture comprising transition layers and dense blocks, as shown in Figure 5. The transition layers (Figure 5a) are connected in the following order: BN layer, ReLU, 3D convolution layer (3 × 1 × 1), and average pooling 3D layer (2 × 2 × 2). The dense blocks (Figure 5b) are organized in the following order: BN layer, ReLU, 3D convolution layer (3 × 1 × 1), BN layer, ReLU, 3D convolution layer, and 3D convolution layers (3 × 3 × 3).

**Figure 6:**
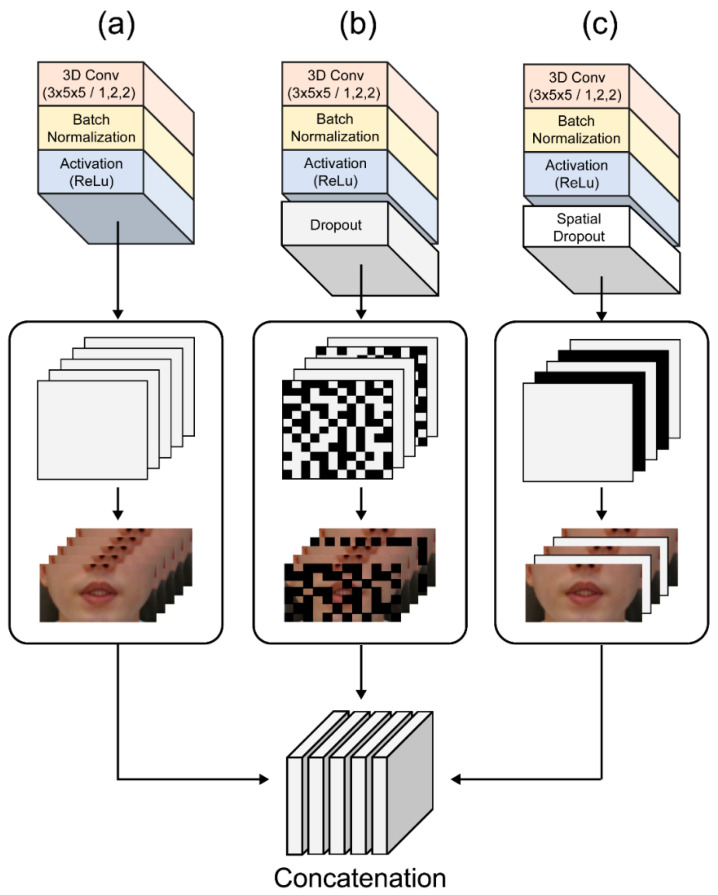
Multilayer 3D CNN architectures: (**a**) first architecture; (**b**) second design with the dropout layer; (**c**) third architecture with the spatial dropout layer.

To date, image classification tasks handled with various CNN models have demonstrated exceptional performance. For example, using the fusion of several CNNs for feature aggregation, it is feasible to extract diverse spatial and temporal information by building different scales and depths [32]. In addition, a different convolutional layer can extract different features for the multilayer 3D CNN training phase to obtain more diverse feature information. Furthermore, by using different depths and filters of varying sizes, multiple features may be created from this training process. Certain associated qualities that were lost in the layered design can be chosen using this strategy, resulting in a richer final feature. The suggested multilayer 3D CNN architecture is shown in Figure 3c. The first module follows the 3D dense connection convolution layer output feature in the order of a 3D convolution layer (64 3D kernels of size 3 × 5 × 5) and then a BN-ReLU layer (Figure 6a). The second module (Figure 6b) includes a dropout layer to prevent overtraining and overfitting that improves and generalizes the CNN’s performance by preventing strongly correlated activations. This is important because of the small size of the benchmark dataset compared to other image datasets [24]. The structure of the third module drops the entire feature map by adding a spatial dropout layer to the structure of the first module (Figure 6c). Unlike the traditional dropout method that removes pixels at random, this method uses CNN models with significant spatial correlation to provide superior picture categorization [33]. As a result, we used a spatial dropout layer to efficiently extract the shape of the lips, teeth, and tongue, which are fine movements around the mouth, with a significant spatial correlation.

### 3.2. Structure of Comparative Feature Extraction Methods

We compared the proposed method to other feature extraction methods, such as LipNet, LeNet-5, CNN Autoencoder, and ResNet-50, which all exhibit an outstanding feature extraction performance. The feature extraction method of LipNet as a baseline comprises 3 × (spatiotemporal convolutions, channel-wise dropout, and spatial max-pooling) [22]. LeNet-5 is the earliest model of deep learning and uses a gradient-based CNN structure for handwritten digital recognition [34]. The input layer of a typical LeNet-5 structure diagram is a handwritten digital image of 0 with a size of 32 × 32, whereas the output layer comprises 10 nodes corresponding to 0. LeNet-5 comprises six layers in total, namely the input and output layers: three convolutional layers, two pooling levels, and one fully connected layer. Convolutional core sizes in the convolutional and pooling layers were set to 5 × 5 and 2 × 2, respectively. However, the training parameters were reduced when the connection layer decreased the number of neurons from 120 to 84. Thus, an unsupervised model that learns to rebuild the input is used as a typical autoencoder [35].

In several domains, such as speech recognition and computer vision, deep learning models can learn intricate hierarchical nonlinear features that can provide superior representations of original data [36]. Encoder, hidden, and decoder layers comprise the autoencoder. The hidden layer’s input is the encoder layer’s output, and the decoder layer’s input is the encoder layer’s output. We created an autoencoder model using LipNet’s feature extraction method for experimental comparison. ResNet-50, a convolutional neural network with 50 layers, is a ResNet [37] version comprising 48 convolution layers, a MaxPool layer, and an average pool layer. The deep residual learning architecture lies at the heart of ResNet. ResNet-50 is substantially smaller than other current designs, with 50 layers and over 23 million trainable parameters; extremely deep neural networks can be employed to circumvent the vanishing gradient problem.

### 3.3. Recurrent Layer

The GRU is one of the recurrent neural networks and is a method of governing and propagating information flow across many time stages [25]. GRUs are derived from LSTM units that determine what information should be carried forward and which should be disregarded. Given that the 3D CNN only captures brief viseme-level data, it may be able to comprehend wider temporal contexts, which is beneficial for ambiguity detection. Because the GRU uses update and reset gates, the gradient vanishing problem can also be overcome. A bi-directional GRU is used as a sequence processing module in the proposed architecture. Compared to typical GRU deployment, a bi-directional GRU provides information in both forward and backward directions to two distinct neural network topologies coupled to the same output layer, allowing both networks to gain full knowledge of the input.

### 3.4. Transcription Layer

We used the CTC method, which employs a loss function to parameterize the distribution of a label token sequence without requiring the alignment of the input sequence to an end-to-end deep neural network. CTC is conditionally independent of the marginal distributions established at each time step of the temporal module as it restricts the usage of autoregressive connections to manage the inter-time-step dependencies of the label sequence. Therefore, CTC models are decoded using a beam search procedure to restore label temporal dependence, and the language model’s probabilities are mixed.

## 4. Experiment

### 4.1. Dataset

We utilized Google Speech Command Dataset v2 and gathered a dataset to analyze the performances of the five OCSR APIs and the proposed model (Supplementary Figure S1a) [38]. Google Speech Command Dataset v2 was released in April 2018, and it contained 105,829,35 word utterances in one second or less. Several experiments have been conducted using this dataset to evaluate the performance of speech recognizers [39,40,41]. In addition, we employed the most frequently used speech recognition command in IoT or real life from the Word Choice part of Google Voice Command Data Set v2 to assess the proposed model [38]. In real-life applications, because an important unit of speech recognition is not the entire sentence but words or short phrases, we selected words useful as commands in automobiles, robot applications, etc. Data were gathered by enlisting people acquainted with speech recognition technology. A total of 40 people (20 males and 20 females with a mean age of 29.14 years) participated, and they were compensated with a $20 voucher. The participants were provided pin microphones to wear, as shown in Supplementary Table S2a. The participants stood at a distance of 1.2 m from the front camera and lighting, and each participant received a list of 20 keywords. The participants repeatedly uttered the same word 100 times at a rate of 2 s per keyword for 2 h; we stored the audio and video information and generated a total of 80,000 videos (Supplementary Table S2b).

Using a Dlib face detector, the targeted area used as the input for the end-to-end lip-reading was detected in the data pre-processing phase by employing a HoG-feature-based linear classifier [23]. The output was subsequently supplied in the form of (x, y) diagonal edge coordinates, which were then used to construct the bounding box around the mouth. Then, using a facial landmark predictor to detect movement around the lips and extract points of lips identical to those obtained from the training dataset, 68 land-marks and online Kalman filters were used in the iBug program [42]. Using an affine transformation, a mouth-centered region with dimensions of 100 × 50 pixels per frame was extracted, and the RGB channels throughout the full training set were normalized to provide zero mean and unit variance. To avoid overfitting, we adopted the data augmentation approach from [22] for the training data. In the training procedure, regular and horizontally mirrored picture sequences were employed. We used individual words as additional training cases with a decay rate of 0.925, considering that the dataset contained the starting and ending terms serving as timers for each “clip” sample to enhance training data at the word level. With a probability of 0.05/frame to eliminate variation, we identified the movement speed and duplicate frames. The same dataset pre-processing and augmentation approaches were used to train and assess all models.

### 4.2. Implementation

We used custom Python scripts to build the OCSR API methods (Python3.6; Rossum, 2019). These scripts were used as wrappers for loading and submitting audio recordings to OCSR API providers and for saving the resulting transcripts. All four providers (Microsoft Azure, Naver Clova, AWS, and Google Cloud) adopted OCSR APIs. While an entire chapter could be transcribed in one instance using Microsoft Azure, Naver Clova, and AWS, only 60 s of the audio per file could be transcribed using Google Cloud. Before analyzing the text, all capitalization and punctuation were removed, and all numbers were converted to text. Our metric for assessing the OCSR API performance was the percentage of correctly transcribed phrases for each recording. In addition, to compare the predicted words to the actual words, the recognition performance was evaluated. If the two words were found to be the same, the recognized word would be true; otherwise, it would be false. The five-word lists following the OSCR API were converted into vectors using the pre-trained Word2Vec model and concatenated into one single vector.

All models of the end-to-end lip-reading architecture were constructed using Keras with a TensorFlow backend and TensorFlow-CTC decoder to evaluate the character accuracy rate (CAR) using the CTC beam search. The complete configuration and parameters utilized for the layers in our proposed architecture are shown in Figure 3 and listed in Supplementary Table S1. All model network parameters were initialized through He initialization, with the exception of square GRU matrices with orthogonal initialization and default hyperparameters. The proposed lip-reading model was trained in the multilayer 3D CNN using channel-wise dropped pixels and the dropped channel utilizing spatial dropout, where the proposed lip-reading architecture was the baseline that was trained on the collected dataset until overfitting. Therefore, our combined proposed system learned using the Adam Optimizer [43] with a learning rate of 0.0001 and mini batches of size 8 by combining the audio and visual vector. The combined proposed system was evaluated in an environment different from that of the data collection, while considering factors affecting accuracy, such as illumination. For the evaluation phase, a person stood 1.5 m away from the KIOSK screen, and a webcam was installed above the screen (Supplementary Figure S1b). The room had normal lightning conditions and controlled noise levels. The evaluation process was divided into three categories to test whether the different components of the system not based on the participants contributed to model training. In the audio-only (A) and visual-only (V) categories, only auditory or visual information was used for recognition, whereas both auditory and visual information were used simultaneously for recognition (multimodal information) in the AV category. None of the participants participated in the data collection phase.

### 4.3. Performance Evaluation Metrics

We employed standard automated voice recognition assessment measures to evaluate the proposed deep-learning model. The learning loss of all models was examined to assess their learning status during the training process. In addition, we evaluated the parameters, training time, and character accuracy rate (CAR) for each model to compare their performances and computational efficiencies. The total edit distance was calculated to convert the error rate measurements, which is used to evaluate accuracy, into percentages. It was important to compare the decoded and original texts for analyzing misclassifications. The CAR percentage is given by

(1) $$\mathrm{CAR}\left(\%\right)=100-\left(\frac{C_{S}+C_{D}+C_{I}}{C_{N}}\right)\times 100,$$

where N, S, I, and D represent the total number of characters in the ground truth, number of characters substituted for incorrect classifications, number of characters inserted for non-picked characters, and number of deletions that should not be present for decoded characters, respectively. As a result, CAR is computed using (N), with C denoting the words. We generated an approximate maximum-probability prediction for all experimental models with the CTC beam search using a Tensor-Flow-CTC decoder. We also analyzed the CAR over the training period in terms of the number of parameters and computational efficiency. To visualize the data, we employed the phoneme-to-viseme mapping approach reported in [44]. The signal-to-noise ratio (SNR) was evaluated by synthesizing noise into the acquired source data. The diverse environments multichannel acoustic noise database (DEMAND) was collected using 16-channel array microphones [45]. For ambient noise, we used different types of noises divided into eight environments (park, hallway, cafeteria, station, café, square, car, and living), and we synthesized noise to evaluate the SNR (Supplementary Table S3). In each sample, the target speech was mixed with eight noises.

## 5. Results

### 5.1. Performance of OCSR APIs

**Figure 7:**
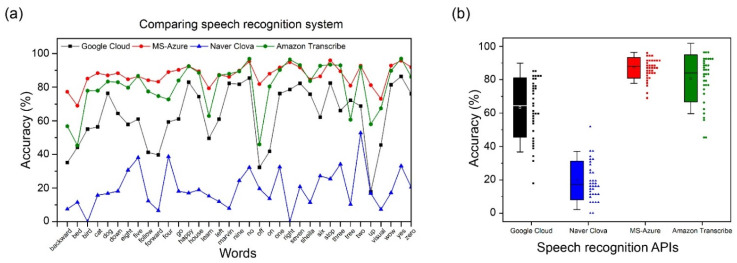
(**a**) Comparison of the performance of speech recognition APIs for 35 individual words in Google dataset V2; (**b**) Comparison of the mean and distribution of speech recognition APIs for Google dataset V2. Error bars represent standard deviation. The squares on the left represent the mean and distribution, and the 35 small structures on the right represent the respective accuracies for 35 words of Google dataset V2.

Figure 7 shows the mean and distribution of the individual and overall performances for all 35 words of the Google Speech Commands Dataset V2 using 5 speech recognizer APIs. Each word has a different number of datasets, and the results for the individual performance are listed in Supplementary Table S4. The performance evaluation was considered to be correct if the word was the same as the prediction result of the recognized data. The Google API shows a low performance for certain words (e.g., forward, off, and up) and a wide variance. Naver shows a low overall performance and has a wide distribution, as shown in Figure 7b. Amazon shows better performance than the other two APIs; however, it demonstrates lower recognition rates for certain words (e.g., off, tree, and up) as well as the Google API. However, among all APIs, Microsoft shows the highest performance for all words, with excellent average and dense distribution results. Thus, Microsoft Azure was selected as the main API.

### 5.2. Training Procedure and Learning Loss

**Figure 8:**
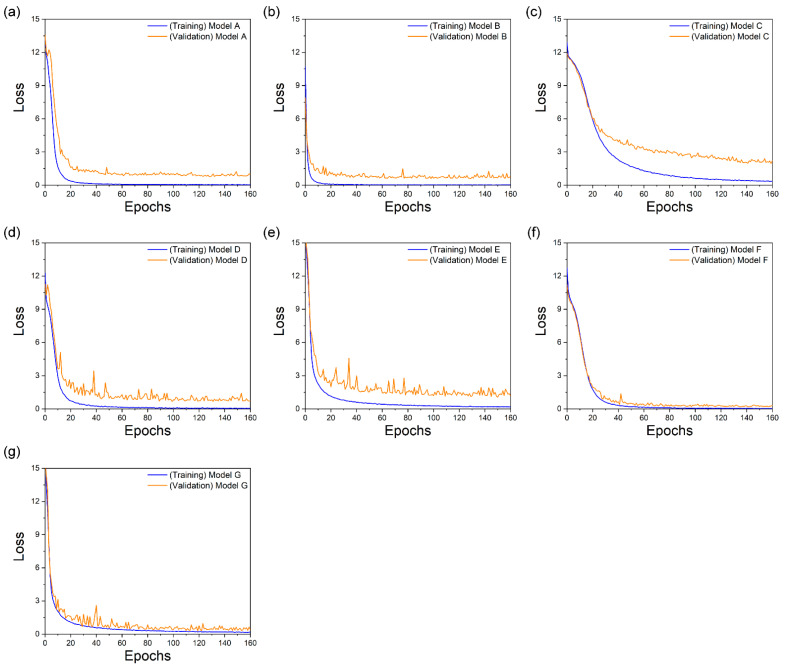
Training and validation loss of the collected dataset. Models (**a**) A; (**b**) B; (**c**) C; (**d**) D; (**e**) E; (**f**) F; and (**g**) G.

Figure 8 shows the training and validation losses that occurred when training the collected dataset with the audio and visual information. For audio, words output from the OCSR API are represented by word vectors using the pre-trained Google’s Word2Vec model. For visual, the seven models have different visual feature extraction modules at the front end and the same sequence processing modules at the back end (Table 1). Model A contained the same architecture as LipNet, the baseline model, whereas Models B, C, D, E, and F used the feature extraction methods of LeNet-5, Autoencoder, ResNet-50, DenseNet-121, and Multilayer CNN, respectively (Section 3.2). The training and validation losses of all seven models were in good agreement. However, the gap between the training and validation losses was the largest in Model C, and its overfitting phenomenon was higher compared to those of other models. Further, while Model F showed lower overfitting results (smallest among all models), it exhibited a lower convergence speed rate than those of Models A, B, D, and E. Therefore, the learning and convergence speeds of Model G (proposed model) were high, and the gap was small. These findings show that the suggested model for the gathered dataset had the smallest difference between training and validation losses, hence preventing overfitting.

### 5.3. Characteristic Accuracy Rate

**Figure 9:**
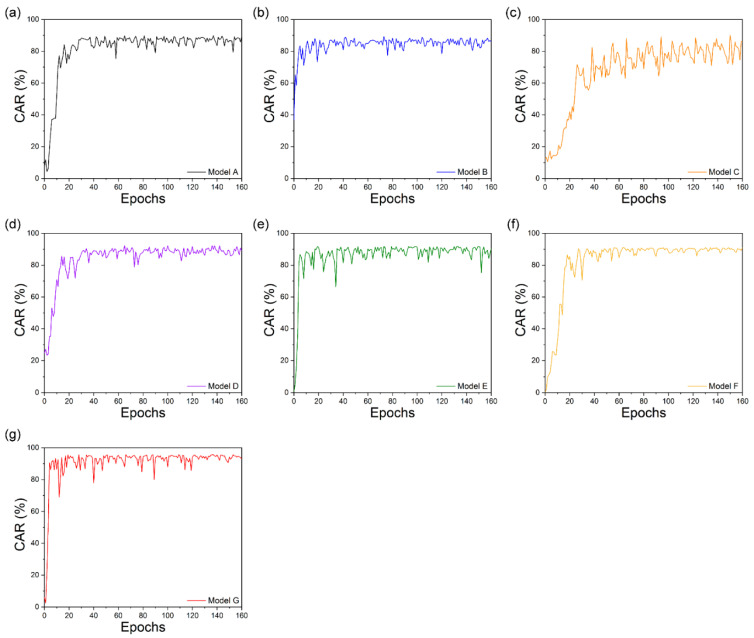
Training steps for CAR comparing the proposed model toother models: Models (**a**) A; (**b**) B; (**c**) C; (**d**) D; (**e**) E; (**f**) F; and (**g**) G.

The results of the comparison between the proposed model and current deep-learning models are listed in Table 1. The suggested model obtained the best results, with a CAR of 95.893%, which was higher than those of the other models and baseline values in all cases. Despite the increase in accuracy of Models (C), (E), (D), and (F) over the baseline, no significant differences were detected (Table 1). Therefore, the proposed model outperformed the existing models, including the baseline model, in terms of accuracy; this could be owing to the combined use of various 3D CNN architectures. Figure 9 compares the proposed model to all other models after training with CAR on the obtained dataset. In addition, a dataset available from [46] was used to assess the performance of the suggested model. The performance of the suggested model remained unchanged, proving its superiority for both open and collected datasets.

### 5.4. Model and Computational Efficiency

**Figure 10:**
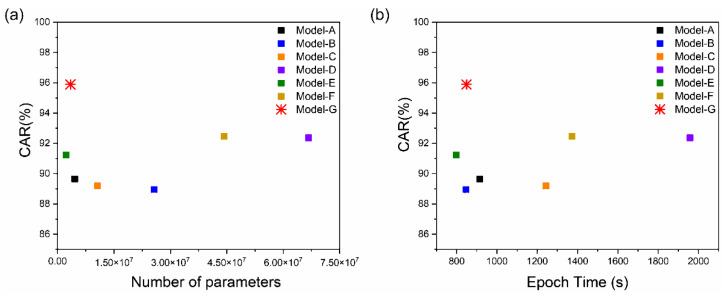
CAR of the baseline and other models according to the (**a**) number of parameters; (**b**) average epoch time.

The model size and computational efficiency of the proposed systems are the main limitations of real-time applications. We examined the accuracy and computational efficiency of the models using varying numbers of trained parameters and training times (Figure 10). Figure 10a shows the performance according to the number of parameters; Figure 10b shows the results of the average training time comparison of the seven models for 160 epochs. Although the proposed model is similar to the baseline model (approximately 50 s), it showed a high CAR while using approximately 11.2 M fewer parameters compared to the other six models that used the collected dataset listed in Table 1. Compared with the baseline model performance, we were able to improve the accuracy while lowering the number of training parameters by approximately 11.2 M and achieving a comparable training time when using our database.

### 5.5. Confusion Matrix

**Figure 11:**
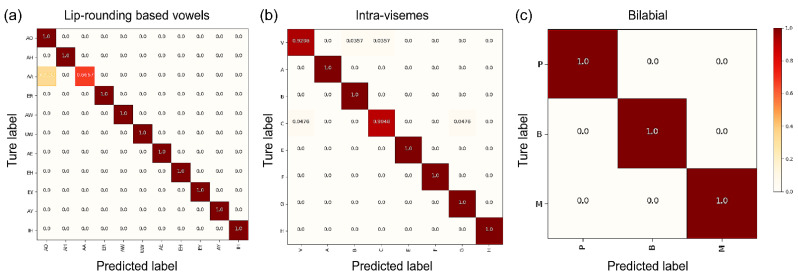
Detailed proposed architecture confusion matrices for the (**a**) lip-rounding based vowels; (**b**) intra-visemes; (**c**) bilabial groups. The three groups with the greatest confusions and confusions inside viseme clusters were selected.

Visual analysis was conducted using IBM ViaVoice database mapping [44]. A confusion matrix was created for the most confusing phoneme in the bilabial viseme class (Figure 11), which included lip-rounding-based vowels, intra-visemes, and bilabial visemes. The experimental results showed that {/AA/, /AO/} is frequently misclassified during the text decoding process (Figure 11a). To produce the vowel sound /AA/, as in “bat”, the mid-back portion of the tongue must be raised, followed by the front and back portions of the tongue stretching in opposite directions. For producing the sound /AO/, as in “orange” and “port”, the tongue and mouth become tighter than that when making the /AA/ sound. The recognized experiment results showed that misclassification generated from “on” and “off” with the shortest duration time with a small mouth opening and incorrect recognition results were obtained from the “er” portion of “center” and “under”. The intra-viseme categorical confusion matrix is shown in Figure 11b. As illustrated in the experimental results for [p], [b], and [m] in Figure 11c, distinguishing homophones was difficult. Based on our assessment of the proposed model considering different perspectives, this model can assist in overcoming technological impediments to practical implementation.

### 5.6. Performance of Combined System

**Figure 12:**
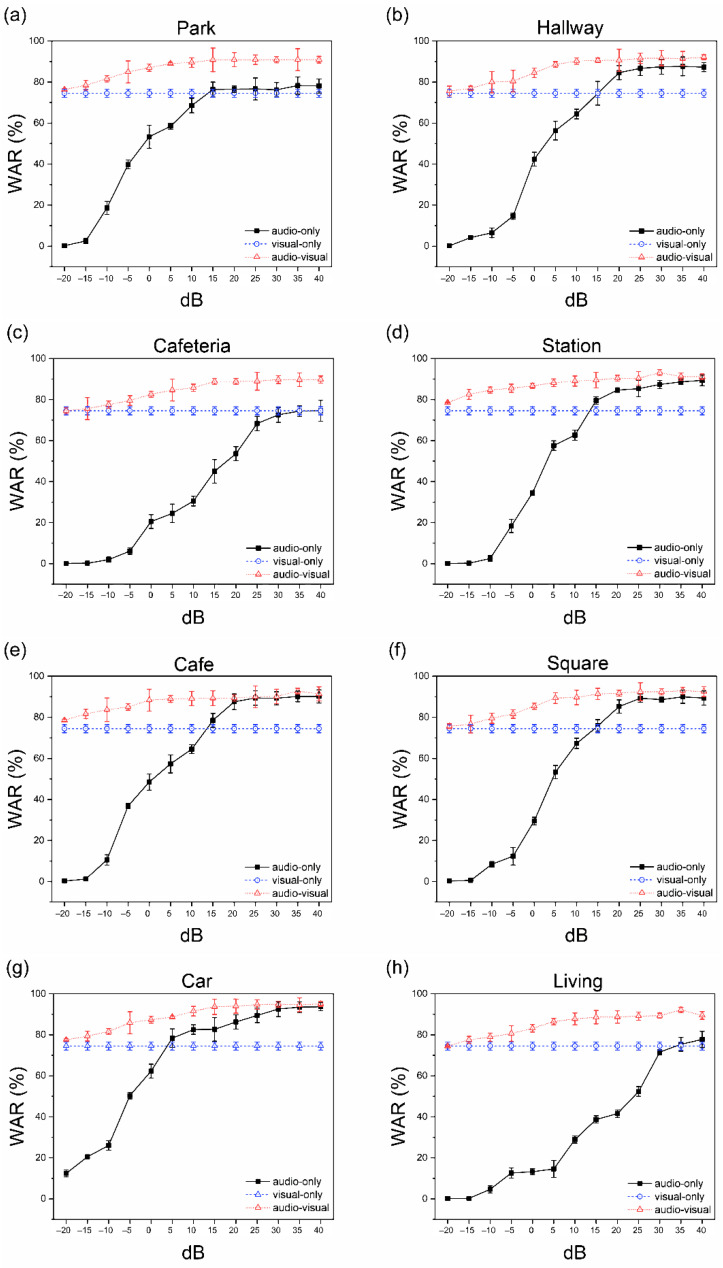
Average recognition accuracy rate with a standard deviation under eight noise environments. Error bars represents standard deviation. The black (single-modal recognition) line represents the audio recognition result, the blue (single-modal recognition) line represents the visual recognition result, and the red (multimodal recognition) line represents the audio-visual recognition result.

Considering the case of park noise in Supplementary Table S5a, we described the WAR for each recognizer (e.g., A, V, and AV) at different SNR levels. For an auditory-only (A) recognizer, the highest word accuracy rate was 78.28% ± 4.21%, which was obtained at an SNR of 35 dB, as shown in Figure 12a. The WAR of visual-only (V) for various SNR values were calculated as 74.54% ± 1.96%. The V recognition cases relied only on visual information and remained unaffected by the clearance of the audio signal or SNR level. Participants in the A, V, and AV recognition cases did not participate in the data collection phase. The process of evaluating A, V, and AV recognition using the KIOSK for performance was different from the data collection environment because the experiment was conducted naturally without limiting variables such as the height of the speaker against the fixed cameras, the shape of the mouth during pronunciation, and lighting. Therefore, different factors could contribute to the difference in the WAR between learning outcomes and evaluation in some real-world applications.

The AV recognition cases, in which both auditory and visual signals were used simultaneously for recognition, exhibited WAR scores of 90.94% ± 1.62%, which showed a 12.66% improvement in the recognition rate compared to A recognition. Therefore, combining sound and images can effectively infer spoken words in the presence of ambient noise.

Based on previous results, we compared the performance of multimodal recognition cases (AV) and single-modal recognition (A) while changing the noise environment. We switched the applied noise environment to a hallway-like noise environment, as listed in Supplementary Table S5b. Thus, the WAR of AV was calculated as 92.09% ± 1.18% with enhancement rates of up to 4.44% compared to the WAR of A.

Supplementary Table S5c,d show the results obtained when the noise environment is switched to the cafeteria and station, respectively. For the cafeteria-like noise environment, the (A) recognition cases scored the lowest value with a WAR of 74.53% ± 5.14%. However, the recognition rate of the AV was 89.76% ± 1.96%, with a 15.23% improvement compared to case A (Figure 12c). In the station noise environment, the AV recognition rate was 93.14% ± 1.51% with an improvement of 3.77% compared to that of A (89.37% ± 2.61%), as shown in Figure 12d.

The street category comprised two different environments: a café and square. The terrace of the café in a public square was considered, and the square was a public town square with many tourists, as listed in Supplementary Table S5e,f, respectively. For the café noise environment, the recognition rates for A and AV were 90.12% ± 3.21%, and 92.78% ± 1.35%, respectively, with an improvement in recognition accuracy up to 2.66% for AV (Figure 12e). For the square noise environment, the recognition rates of A and AV were 89.96% ± 3.18%, and 92.93% ± 1.73%, respectively, with an improvement in recognition accuracy of up to 2.97% for AV.

The environment in which audio-only recognition (A) had the highest recognition rate was cars (Figure 12g and Supplementary Table S5g). The recognition rates for A and AV were 93.68 ± 2.03%, and 95.01% ± 1.18%, respectively. The recognition accuracy for AV was up to 1.33%.

For the living room noise listed in Supplementary Table S5h, the recognition rates of A and AV were 77.71% ± 3.94% and 92.13% ± 1.35%, respectively. For AV, the recognition rates were improved by 14.42% (Figure 12h).

**Figure 13:**
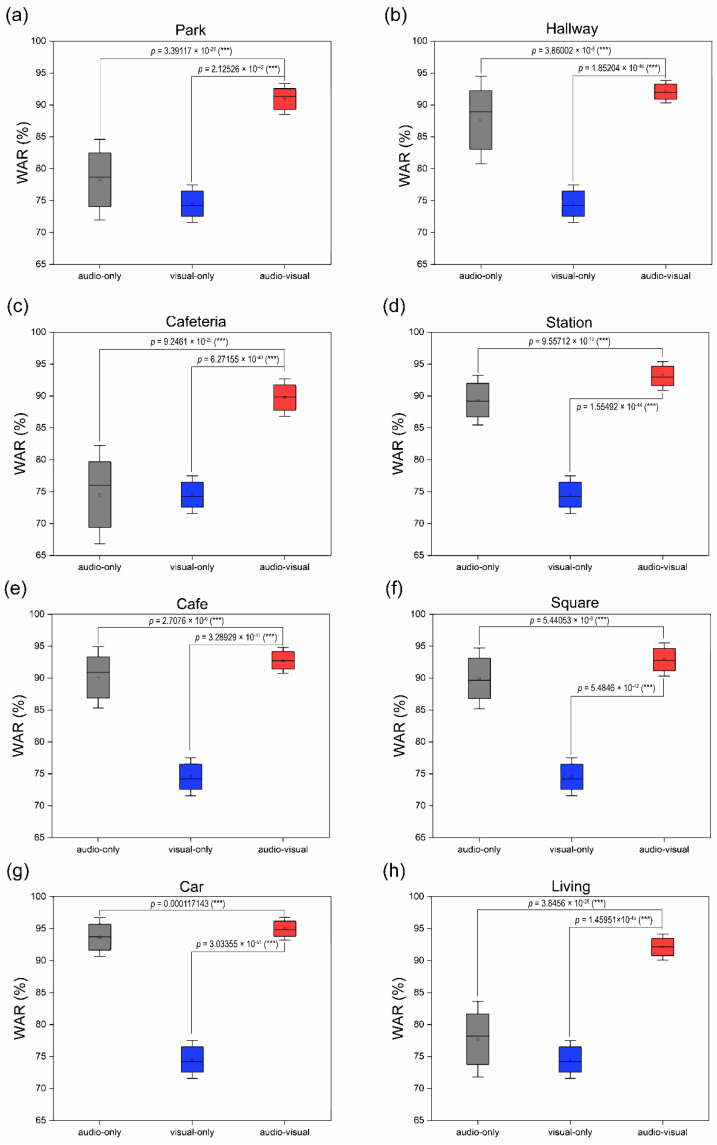
Best average recognition accuracy rates under eight noise environments. Error bars represent standard deviation. Asterisks represent statistical significance-based *t*-tests between each group (* for *p* < 0.05, ** for *p* < 0.01, *** for *p* < 0.001). The gray (single-modal recognition) line represents the audio recognition result, the blue (single-modal recognition) line represents the visual recognition result, and the red (multimodal recognition) line represents the audio-visual recognition result.

Figure 13 shows the statistical significance of the t-test between each group. In general, AV showed an average improvement in the recognition rate of 11.05% and 7.19%, respectively, compared to A. Supplementary Table S6 lists a significant difference of 20.48% between the highest (95.01%) and lowest (74.53%) recognition rates across all eight environments. However, the difference between the maximum and minimum was significantly reduced from 19.15% (A (difference of cafeteria and car)) to 5.251.93% (AV, (cafeteria and car)) by combining multimodal inputs (audio and visual) to recognize spoken words. Thus, unlike A, which showed a good recognition rate in a specific environment (e.g., car noise environment), AV exhibited a superior recognition rate in multiple noise environments (eight environments).

## 6. Discussion

In recent years, the ASR technology has significantly improved because of the exponential increase in large data and processing power, which has made it possible to create complex applications such as voice search and interactions with mobile devices, voice control in home entertainment systems, and various speech-centric information processing applications that benefit from the downstream processing of ASR outputs [3]. Considering that OCSR APIs must function appropriately in demanding acoustic scenarios compared to those in the past, noise robustness has become an essential core technology for large-scale, real-world applications.

This study proposes noise-robust OCSR APIs based on an end-to-end lip-reading architecture for practical applications in various environments. We compared the performance of five OCSR APIs with excellent performance ability. Among all OCSR APIs, Microsoft’s API achieved the best performance on the Google Speech Command Dataset V2. Further, we evaluated the performance of several deep-learning models that analyzed visual information to predict keyword sequences. The results show that the proposed architecture achieves the best performance. Moreover, the proposed system requires fewer parameters and provides faster training times than those of the existing models. Compared to the baseline model, the proposed model decreased the number of parameters by 11.2 M and increased the accuracy by 6.239%.

We measured the SNR of the combined proposed system by synthesizing eight noise data and OCSR API outputs to compare the performance for various noise environments. Audio-based speech recognition systems, which showed excellent performance in only specific environment such as a car, demonstrated stable and excellent performance in all environments using visual information. Supplementary Table S6 shows the highest word accuracy and standard deviation values for each of the eight environments. The lowest and highest recognition rates of audio-based speech recognition were calculated as 74.53% and 95.01%, respectively, with a difference of 19.15%, which indicates a significant performance difference based on the specific environment. However, the difference between the two performances was reduced from 19.15% to 5.25% by adding visual information using multimodal interaction methods, and the same performance was achieved in several environments. To solve problems based on the type of environment, two sets of experiments (A, V, and AV) were conducted; stable performances were observed in all environments. The proposed system showed consistent performance in various environments compared to the performance of conventional audio-based speech recognition, which showed excellent performance in only specific environments.

## 7. Conclusions

We demonstrated a speech recognition system robust to noise using multimodal interaction based on visual information. Our system consisted of an architecture that combines audio and visual information, and its performance was evaluated under eight noise environments. Unlike conventional speech recognition, which shows high performance only in specific environments, we showed the same stable high performance in various noise environments, and simultaneously showed that visual information contributed to improving speech recognition. Therefore, our method showed a stable and high performance in various noise environments by combining lip-reading, a technology that can enhance the speech recognition system, with existing cloud-based speech recognition systems. This system has potential in various applications, such as IoT and robot applications, that use speech recognition in noise and can be useful in various real-life applications where speech recognition is frequently used, particularly indoors, including hallways, cars, and stations, and outdoors, such as parks, cafés, and squares.

### Future Work

Multimodal interactions based on visual information must be used to produce noise-resistant ASR. The proposed system may be helpful for patients who have difficulty in conversation owing to problems with speech recognition in noisy environments. However, applying this technology to conversation recognition is problematic. Therefore, we will seek to expand the system’s capabilities to identify phrases rather than individual words in the future. We will also examine the performance of the suggested system in real-world settings involving humans and machines. Despite the intense effort invested into the development of an accurate speech recognition system, the development of a lightweight system that is robust with respect to real-life circumstances while accounting for all uncertainties is still challenging.

## Figures and Tables

**Table 1:** Performance, number of parameters, and training time of proposed model compared to those of the baseline and other models.

Model	Audio	Vision		Parameters	Average Epoch Time (s)	CAR (%)
		Front-End	Back-End			
A	Google’s Pertained model	3D CNN	Bi-direction GRU + CTC	4,571,388	914.74	89.654
B		3D CNN + 3D LeNet-5		3,651,038	847.18	88.948
C		3D CNN + Autoencoder		10,576,269	1242.89	89.189
D		3D CNN + 3D ResNet-50		66,705,692	1957.81	92.365
E		3D CNN + 3D DenseNet-121		2,247,537	798.77	91.237
F		3D CNN + Multilayer 3D CNN		44,327,404	1372.30	92.459
G		Proposed architecture		3,455,857	849.44	95.893

## Data Availability

The data presented in this study are available on request from the corresponding author. The data are not publicly available due to ethical restrictions. The database used in this article is Google Speech Commands Dataset V2. For details, please refer to [38].

## Supplementary Materials

The following supporting information can be downloaded at: https://www.mdpi.com/article/10.3390/s22082938/s1, Figure S1. (a) Data recording environment for audiovisual data and (b) evaluation of auditory-visual speech recognition system; Table S1. Hyperparameters of the proposed CNN architecture; Table S2. (a) Google Speech Commands Dataset v2 and (b) Collected Speech Commands Dataset; Table S3. Noise database structure: Categories and recordings conducted in each category; Table S4. Performance evaluation of speech recognition systems on Google Speech Commands Dataset V2; Table S5. Average word accuracy and standard deviation of proposed system in eight environments; Table S6. Best word accuracy and standard deviation of proposed system in eight environments.
