# Supplementary material for: End-to-End Lip-Reading Open Cloud-Based Speech Architecture
Source: Sensors (Basel). 2022 Apr 12;22(8):2938. doi: 10.3390/s22082938 (PMC9029225; doi:10.3390/s22082938)
Supplement: Supplementary file 1 [file sensors-22-02938-s001.zip › sensors-1622271-supplementary.pdf]

sensors MDPI

Article

Supplementary materials

End-to-End Lip-Reading Open Cloud-Based Speech Architecture

Sanghun Jeon and Mun Sang Kim *

Center for Healthcare Robotics, Gwangju Institute of Science and Technology (GIST), School of Integrated Technology, Gwangju 61005, Korea; jeon7887@gist.ac.kr

* Correspondence: munsang@gist.ac.kr; Tel.: +82-10-9126-4628

Citation: Citation: Jeon, S.; Kim, M.S. End-to-End Lip-Reading Open Cloud-Based Speech Architecture. *Sensors* **2022**, *22*, 2938. https://doi.org/10.3390/2938

Academic Editors: Bruce Denby, Tamás Gábor Csapó, Michael Wand and Nikolaos Doulamis

Received: 17 February 2022
Accepted: 8 April 2022
Published: 12 April 2022

Publisher's Note: MDPI stays neutral with regard to jurisdictional claims in published maps and institutional affiliations.

Copyright: © 2022 by the authors. Submitted for possible open access publication under the terms and conditions of the Creative Commons Attribution (CC BY) license (https://creativecommons.org/licenses/by/4.0/).

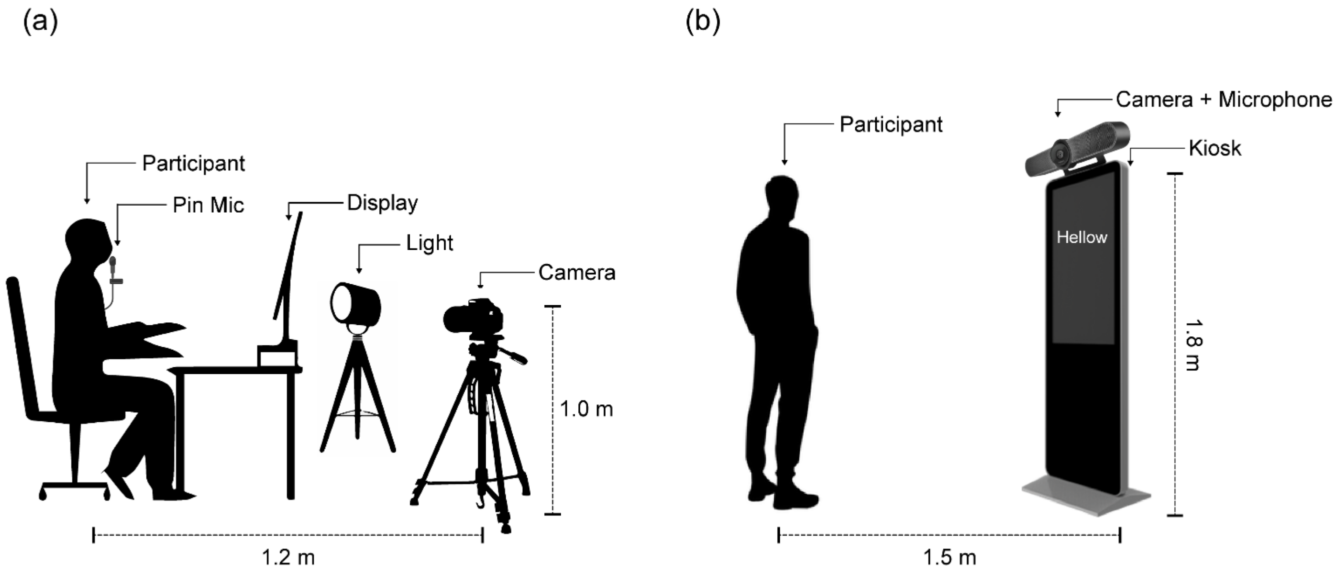

Supplementary Figure S1. (**a**) Data recording environment for audiovisual data and (**b**) evaluation of auditory-visual speech recognition system.

Supplementary Table S1. Hyperparameters of the proposed architecture.

**Layers**	**Size / Strid / Pad**		**Visual**	**Audio**	
			**Output Size**		**Dimension Order**
3D Conv	[3 × 5 × 5] / (1, 2, 2) / (1, 2, 2)		60 × 50 × 25 × 64		T × C × H × W
3D Max Pooling	[1 × 2 × 2] / (1, 2, 2)		60 × 50 × 13 × 64		T × C × H × W
3D Dense Block (1)	[3 × 1 × 1] 3D Conv	(×6)	60 × 25 × 13 × 96		T × C × H × W
	[3 × 3 × 3] 3D Conv				
3D Transition Block (1)	[3 × 1 × 1] 3D Conv		60 × 12 × 6 × 6		T × C × H × W
	[1 × 2 × 2] average pool / (1 × 2 × 2)				
3D Dense Block (2)	[3 × 1 × 1] 3D Conv	(×12)	60 × 12 × 6 × 38		T × C × H × W
	[3 × 3 × 3] 3D Conv				
3D Transition Block (2)	[3 × 1 × 1] 3D Conv		60 × 6 × 3 × 3		T × C × H × W
	[1 × 2 × 2] average pool / (1 × 2 × 2)				
3D Dense Block (3)	[3 × 1 × 1] 3D Conv	(×24)	60 × 12 × 6 × 38		T × C × H × W
	[3 × 3 × 3] 3D Conv				
3D Transition Block (3)	[3 × 1 × 1] 3D Conv		60 × 3 × 1 × 1		T × C × H × W
	[1 × 2 × 2] average pool / (1 × 2 × 2)				
3D Dense Block (4)	[3 × 1 × 1] 3D Conv	(×16)	60 × 3 × 1 × 33		T × C × H × W
	[3 × 3 × 3] 3D Conv				
Multilayer 3D CNN (1)	[3 × 5 × 5] / (1, 2, 2) / (1, 2, 2)		60 × 3 × 1 × 64		T × C × H × W
Multilayer 3D CNN (2)	[3 × 5 × 5] / (1, 2, 2) / (1, 2, 2)		60 × 3 × 1 × 64		T × C × H × W
Multilayer 3D CNN (3)	[3 × 5 × 5] / (1, 2, 2) / (1, 2, 2)		60 × 3 × 1 × 64		T × C × H × W
Bi-GRU (1)	256		60 × 512		T × F
Bi-GRU (2)	256		60 × 512	1500	T × F
Concatenation			60 × 2012		T × F
Linear	27 + blank		60 × 2012		T × F
Softmax			60 × 28		T × V

Supplementary Table S2. (**a**) Google Speech Commands Dataset v2 and (**b**) Collected Speech Commands Dataset.

(**a**) Google Speech Commands Dataset v2

backward	bed	bird	cat	dog
down	eight	five	follow	forward
four	go	happy	house	learn
left	marvin	nine	no	off
on	one	right	seven	sheila
six	stop	three	tree	two
up	visual	wow	yes	zero

(**b**) Collected Speech Commands Dataset

Up	down	play	stop	Left
right	back	next	on	Off
pause	start	turn	center	Under

internet	music	weather	camera	Time

Supplementary Table S3. Noise database structure: Categories and recordings conducted in each category.

	Category	Place	Environment
(a)	Nature	Park	Well-visited city park
(b)	Office	Hallway	Hallway inside an office building, with individuals and groups passing by occasionally
(c)	Public	Cafeteria	Busy office cafeteria
(d)		Station	Main transfer area of a busy subway station
(e)	Street	Cafe	Terrace of a cafe at a public square
(f)		Square	Public town square with many tourists
(g)	Transportation	Car	Private passenger vehicle
(h)	Domestic	Living	Inside a living room

Supplementary Table S4. Performance evaluation of speech recognition systems on Google Speech Commands Dataset V2.

Class	Number of files	Google Cloud		MS-Azure		Naver Clova		Amazon Transcribe	
		Correct	Accuracy	Correct	Accuracy	Correct	Accuracy	Correct	Accuracy
backward	1,664	586	35.22%	1,286	77.28%	125	7.51%	946	56.85%
bed	2,014	891	44.24%	1,390	69.02%	234	11.62%	914	45.38%
bird	2,064	1,137	55.09%	1,757	85.13%	0	0.00%	1609	77.96%
cat	2,031	1,147	56.47%	1,795	88.38%	320	15.76%	1584	77.99%
dog	2,128	1,627	76.46%	1,853	87.08%	359	16.87%	1774	83.36%
down	3,917	2,523	64.41%	3,461	88.36%	714	18.23%	3251	83.00%
eight	3,787	2,193	57.91%	3,208	84.71%	1,163	30.71%	3021	79.77%
five	4,052	2,475	61.08%	3,505	86.50%	1,547	38.18%	3516	86.77%
follow	1,579	652	41.29%	1,329	84.17%	196	12.41%	1224	77.52%
forward	1,557	619	39.76%	1,296	83.24%	103	6.62%	1164	74.76%
four	3,728	2,213	59.36%	3,320	89.06%	1,446	38.79%	2713	72.77%
go	3,880	2,372	61.13%	3,504	90.31%	705	18.17%	3261	84.05%
happy	2,054	1,705	83.01%	1,902	92.60%	351	17.09%	1899	92.45%
house	2,113	1,572	74.40%	1,890	89.45%	402	19.03%	1873	88.64%
learn	1,575	782	49.65%	1,250	79.37%	240	15.24%	991	62.92%
left	3,801	2,318	60.98%	3,322	87.40%	457	12.02%	3313	87.16%
marvin	2,100	1,729	82.33%	1,810	86.19%	167	7.95%	1848	88.00%
nine	3,934	3,217	81.77%	3,536	89.88%	964	24.50%	3519	89.45%
no	3,941	3,368	85.46%	3,759	95.38%	1,275	32.35%	3819	96.90%
off	3,745	1,212	32.36%	3,066	81.87%	737	19.68%	1724	46.03%
on	3,845	1,610	41.87%	3,385	88.04%	529	13.76%	3094	80.47%
one	3,890	2,966	76.25%	3,566	91.67%	1,268	32.60%	3513	90.31%
right	3,778	2,971	78.64%	3,586	94.92%	0	0.00%	3646	96.51%
seven	3,998	3,293	82.37%	3,670	91.80%	834	20.86%	3724	93.15%
sheila	2,022	1,535	75.91%	1,710	84.57%	233	11.52%	1693	83.73%
six	3,860	2,399	62.15%	3,337	86.45%	1,052	27.25%	3581	92.77%
stop	3,872	3,195	82.52%	3,718	96.02%	988	25.52%	3618	93.44%
three	3,727	2,465	66.14%	3,335	89.48%	1,277	34.26%	3467	93.02%
tree	1,759	1,271	72.26%	1,424	80.96%	182	10.35%	1068	60.72%

two	3,880	2,669	68.79%	3,599	92.76%	2,050	52.84%	3574	92.11%
up	3,723	665	17.86%	3,024	81.22%	628	16.87%	2161	58.04%
visual	1,592	727	45.67%	1,164	73.12%	117	7.35%	1074	67.46%
wow	2,123	1,730	81.49%	1,972	92.89%	365	17.19%	1906	89.78%
yes	4,044	3,496	86.45%	3,874	95.80%	1,344	33.23%	3924	97.03%
zero	4,052	3,082	76.06%	3,724	91.91%	832	20.53%	3492	86.18%

sensors MDPI

Supplementary Table S5. Average word accuracy and standard deviation of proposed system in eight environments.

SNR (dB)		−20	−15	−10	−5	0	5	10	15	20	25	30	35	40
(a) Park	A	0.21% ± 0.11%	2.54% ± 1.35%	18.64% ± 3.24%	39.84% ± 2.13%	53.35% ± 5.65%	58.46% ± 1.54%	68.53% ± 3.52%	76.35% ± 3.64%	76.54% ± 1.53%	76.64% ± 5.35%	76.23% ± 3.53%	78.28% ± 4.21%	78.09% ± 3.45%
	V	74.54% ± 1.96%	74.54% ± 1.96%	74.54% ± 1.96%	74.54% ± 1.96%	74.54% ± 1.96%	74.54% ± 1.96%	74.54% ± 1.96%	74.54% ± 1.96%	74.54% ± 1.96%	74.54% ± 1.96%	74.54% ± 1.96%	74.54% ± 1.96%	74.54% ± 1.96%
	A V	76.34% ± 0.53%	78.43% ± 2.35%	81.64% ± 1.53%	84.95% ± 5.35%	86.99% ± 1.74%	89.01% ± 0.53%	89.53% ± 2.35%	90.83% ± 5.74%	90.88% ± 3.45%	90.89% ± 2.35%	90.91% ± 1.53%	90.92% ± 5.35%	90.94% ± 1.62%
(b) Hallway	A	0.19% ± 0.11%	4.23% ± 0.53%	6.51% ± 2.35%	14.66% ± 1.53%	42.43% ± 3.35%	56.31% ± 4.53%	64.41% ± 2.34%	74.52% ± 5.74%	84.56% ± 3.45%	86.67% ± 3.52%	87.45% ± 3.64%	87.65% ± 4.58%	87.28% ± 2.23%
	V	74.54% ± 1.96%	74.54% ± 1.96%	74.54% ± 1.96%	74.54% ± 1.96%	74.54% ± 1.96%	74.54% ± 1.96%	74.54% ± 1.96%	74.54% ± 1.96%	74.54% ± 1.96%	74.54% ± 1.96%	74.54% ± 1.96%	74.54% ± 1.96%	74.54% ± 1.96%
	A V	75.61% ± 2.35%	76.89% ± 1.11%	79.98% ± 5.23%	80.42% ± 5.31%	84.53% ± 2.31%	88.59% ± 1.35%	90.24% ± 1.53%	90.58% ± 1.11%	90.64% ± 5.35%	91.58% ± 2.53%	91.65% ± 3.74%	91.43% ± 3.45%	92.09% ± 1.18%
(c) Cafeteria	A	0.14% ± 0.12%	0.24% ± 0.14%	1.95% ± 1.01%	6.14% ± 1.53%	20.53% ± 3.35%	24.53% ± 4.53%	30.53% ± 2.34%	45.02% ± 5.74%	53.64% ± 3.45%	68.31% ± 3.52%	72.54% ± 3.64%	74.35% ± 2.64%	74.53% ± 5.14%
	V	74.54% ± 1.96%	74.54% ± 1.96%	74.54% ± 1.96%	74.54% ± 1.96%	74.54% ± 1.96%	74.54% ± 1.96%	74.54% ± 1.96%	74.54% ± 1.96%	74.54% ± 1.96%	74.54% ± 1.96%	74.54% ± 1.96%	74.54% ± 1.96%	74.54% ± 1.96%
	A V	74.58% ± 1.53%	75.59% ± 5.35%	77.53% ± 1.74%	79.54% ± 2.35%	82.57% ± 1.53%	84.62% ± 5.35%	85.78% ± 1.74%	88.82% ± 1.52%	88.82% ± 1.53%	88.95% ± 4.35%	89.76% ± 1.96%	89.65% ± 3.35%	89.76% ± 1.74%
(d) Station	A	0.17% ± 0.12%	0.23% ± 0.18%	2.54% ± 1.35%	18.43% ± 3.24%	34.53% ± 1.11%	57.59% ± 2.35%	62.64% ± 2.53%	79.53% ± 1.89%	84.64% ± 1.09%	85.35% ± 3.93%	87.35% ± 1.95%	88.64% ± 1.35%	89.37% ± 2.614%
	V	74.54% ± 1.96%	74.54% ± 1.96%	74.54% ± 1.96%	74.54% ± 1.96%	74.54% ± 1.96%	74.54% ± 1.96%	74.54% ± 1.96%	74.54% ± 1.96%	74.54% ± 1.96%	74.54% ± 1.96%	74.54% ± 1.96%	74.54% ± 1.96%	74.54% ± 1.96%
	A V	78.54% ± 0.23%	82.53% ± 2.42%	84.63% ± 1.53%	85.53% ± 1.98%	86.69% ± 1.25%	88.23% ± 1.89%	89.01% ± 2.45%	89.52% ± 3.86%	90.42% ± 1.47%	90.35% ± 3.35%	93.14% ± 1.51%	91.23% ± 1.74%	91.12% ± 1.35%
(e) Cafe	A	0.23% ± 0.12%	1.24% ± 0.95%	10.53% ± 2.54%	36.92% ± 1.23%	48.53% ± 3.97%	57.35% ± 4.42%	64.53% ± 2.11%	78.46% ± 3.35%	87.53% ± 3.85%	89.35% ± 3.52%	89.24% ± 3.17%	90.11% ± 2.63%	90.12% ± 3.21%
	V	74.54% ± 1.96%	74.54% ± 1.96%	74.54% ± 1.96%	74.54% ± 1.96%	74.54% ± 1.96%	74.54% ± 1.96%	74.54% ± 1.96%	74.54% ± 1.96%	74.54% ± 1.96%	74.54% ± 1.96%	74.54% ± 1.96%	74.54% ± 1.96%	74.54% ± 1.96%
	A V	78.53% ± 0.53%	81.56% ± 2.35%	83.63% ± 5.74%	85.01% ± 1.53%	88.32% ± 5.35%	88.92% ± 1.74%	89.12% ± 3.52%	89.23% ± 3.64%	89.43% ± 1.53%	89.95% ± 5.35%	90.12% ± 3.53%	92.78% ± 1.35%	91.34% ± 3.43%

(f) Square	A	0.23% ± 0.22%	0.56% ± 0.23%	8.42% ± 1.34%	12.34% ± 4.25%	29.52% ± 1.86%	53.35% ± 3.24%	67.35% ± 2.52%	75.83% ± 2.98%	85.23% ± 3.23%	89.24% ± 2.06%	88.53% ± 1.24%	89.96% ± 3.18%	89.32% ± 3.45%
	V	74.54% ± 1.96%	74.54% ± 1.96%	74.54% ± 1.96%	74.54% ± 1.96%	74.54% ± 1.96%	74.54% ± 1.96%	74.54% ± 1.96%	74.54% ± 1.96%	74.54% ± 1.96%	74.54% ± 1.96%	74.54% ± 1.96%	74.54% ± 1.96%	74.54% ± 1.96%
	A V	75.66% ± 1.11%	76.73% ± 4.25%	79.52% ± 2.44%	81.56% ± 2.11%	85.34% ± 1.53%	89.34% ± 2.64%	89.59% ± 3.55%	91.32% ± 2.78%	91.74% ± 1.41%	92.43% ± 4.35%	92.42% ± 1.53%	92.93% ± 1.73%	92.54% ± 2.42%
(g) Car	A	12.42% ± 1.74%	20.53% ± 0.53%	26.06% ± 2.35%	50.34% ± 1.53%	62.30% ± 3.35%	78.35% ± 4.53%	82.54% ± 2.34%	82.64% ± 5.74%	86.24% ± 3.45%	89.46% ± 3.52%	92.54% ± 3.64%	93.46% ± 2.64%	93.68% ± 2.03%
	V	74.54% ± 1.96%	74.54% ± 1.96%	74.54% ± 1.96%	74.54% ± 1.96%	74.54% ± 1.96%	74.54% ± 1.96%	74.54% ± 1.96%	74.54% ± 1.96%	74.54% ± 1.96%	74.54% ± 1.96%	74.54% ± 1.96%	74.54% ± 1.96%	74.54% ± 1.96%
	A V	77.53% ± 0.53%	79.36% ± 2.35%	81.63% ± 1.53%	85.89% ± 5.35%	87.35% ± 1.74%	88.64% ± 0.53%	91.53% ± 2.35%	93.64% ± 3.74%	93.99% ± 3.45%	94.74% ± 2.35%	94.89% ± 1.53%	94.63% ± 3.35%	95.01% ± 1.18%
(h) Living	A	0.22% ± 0.14%	0.23% ± 0.17%	4.65% ± 1.74%	12.59% ± 2.44%	13.25% ± 1.53%	14.56% ± 4.14%	28.92% ± 1.74%	38.71% ± 1.77%	41.53% ± 1.85%	52.35% ± 2.35%	71.54% ± 1.24%	75.35% ± 3.35%	77.71% ± 3.94%
	V	74.54% ± 1.96%	74.54% ± 1.96%	74.54% ± 1.96%	74.54% ± 1.96%	74.54% ± 1.96%	74.54% ± 1.96%	74.54% ± 1.96%	74.54% ± 1.96%	74.54% ± 1.96%	74.54% ± 1.96%	74.54% ± 1.96%	74.54% ± 1.96%	74.54% ± 1.96%
	A V	74.45% ± 0.13%	77.56% ± 1.64%	78.89% ± 2.03%	80.65% ± 3.77%	83.15% ± 1.84%	86.45% ± 1.57%	87.75% ± 2.87%	88.59% ± 3.24%	88.68% ± 3.02%	89.11% ± 2.01%	89.31% ± 1.22%	92.13% ± 1.35%	89.42% ± 1.88%

sensors

MDPI

Supplementary Table S6. Best word accuracy and standard deviation of proposed system in eight environments.

Category	Park	Hallway	Cafeteria	Station	Cafe	Square	Car	Living
A	78.28% ± 4.21%	87.65% ± 4.58%	74.53% ± 5.14%	89.37% ± 2.61%	90.12% ± 3.21%	89.96% ± 3.18%	93.68% ± 2.03%	77.71% ± 3.94%
V	74.54% ± 1.96%	74.54% ± 1.96%	74.54% ± 1.96%	74.54% ± 1.96%	74.54% ± 1.96%	74.54% ± 1.96%	74.54% ± 1.96%	74.54% ± 1.96%
AV	90.94% ± 1.62%	92.09% ± 1.18%	89.76% ± 1.96%	93.14% ± 1.51%	92.78% ± 1.35%	92.93% ± 1.73%	95.01% ± 1.18%	92.13% ± 1.35%
